# SUICIDE IN MILD COGNITIVE IMPAIRMENT [MCI]: DATA FROM OVER 370,000 OLDER VETERANS WITH MCI

**DOI:** 10.1093/geroni/igae098.1132

**Published:** 2024-12-31

**Authors:** Ruth Morin, Aaron Ritter, Yixia Li, Amy Byers

**Affiliations:** Hoag Memorial Hospital Presbyterian, Newport Beach, California, United States; Hoag Memorial Hospital Presbyterian, Newport Beach, California, United States; NCIRE, San Francisco, California, United States; San Francisco VA Healthcare System, San Francisco, California, United States

## Abstract

Prior research has demonstrated that receiving an MCI diagnosis increases risk for near-term suicide attempt. Moreover, psychiatric comorbidity associated with MCI, which may exacerbate conversion to dementia, is also associated with suicide risk. To our knowledge, the current study is the first to comprehensively characterize the occurrence of suicide attempt and associated clinical factors in a large nationally representative sample of older Veterans with MCI. This study leveraged a cohort of Veterans over age 50 who used VA healthcare between 2012-2020 (N = 5,059,526). There were 370,546 individuals with clinically diagnosed MCI identified. Among those, 5,101 attempted suicide (fatal and non-fatal) during the study period (1.4% of the MCI sample). Of those who attempted suicide, 574 were fatal (11.3%). Individuals who attempted suicide were significantly younger, more likely to be female, and less likely to be White than those who did not attempt (all p<.001). Psychiatric comorbidity was strikingly high among the MCI group with a suicide attempt (98.4% with an attempt had at least one psychiatric comorbidity compared to 81.5% who did not attempt). Specifically, there was significantly higher prevalence of any mood disorder, anxiety disorder, substance abuse disorder, psychotic spectrum disorders, and sleep disturbances. Of individuals with MCI who attempted suicide, 71.4% had prior suicide attempt before receiving an MCI diagnosis and nearly 80% had reported suicidal ideation at some point during the study period. Findings indicate a call for investigation into mechanisms of suicide risk and strategies for prevention/intervention in this vulnerable population of adults with MCI.

